# Prospective isolation and characterization of committed and multipotent progenitors from immortalized mouse mammary epithelial cells with morphogenic potential

**DOI:** 10.1186/bcr2863

**Published:** 2011-04-05

**Authors:** Frances S Kittrell, Martha Z Carletti, Sofia Kerbawy, Jessica Heestand, Wa Xian, Mei Zhang, Heather L LaMarca, Arnoud Sonnenberg, Jeffrey M Rosen, Daniel Medina, Fariba Behbod

**Affiliations:** 1 Department of Molecular and Cellular Biology, Baylor College of Medicine, One Baylor Plaza, Houston, TX 77030, USA; 2 Department of Pathology and Laboratory Medicine, University of Kansas Medical Center, 3901 Rainbow Boulevard, Kansas City, KS 66160, USA; 3 Institute of Medical Biology, 8A Biomedical Grove, 06-06 Immunos, 138648, Singapore; 4 Division of Cell Biology, Netherlands Cancer Institute, Plesmanlaan 121, Amsterdam, NL-1066CX, The Netherlands

## Abstract

**Introduction:**

Utilizing single-cell cloning of the COMMA-D cell line engineered to express β-galactosidase (CDβ) cell line, which exhibits normal *in vivo *morphogenesis, distinct multipotent, ductal-limited, alveolar-limited and luminal-restricted progenitors, have been isolated and characterized.

**Methods:**

A single-cell suspension of CDβ cells was stained using Hoechst dye 33342, followed by analysis and sorting. Cells that effluxed the dye appeared on the left side of a FACS analysis panel and were referred to as side population (SP) cells. Cells that retained the dye appeared on the right side and were referred to as non-SP (NSP) cells. Cells from both SP and NSP regions were sorted and analyzed for outgrowth potential. Additionally, individual clones were derived from single cells sorted from each region.

**Results:**

There was no difference in the outgrowth potential of the SP vs. NSP cells when 5,000 cells per fat pad were transplanted. However, individual clones derived from single cells sorted from either SP or NSP regions had varying growth potential. A total of nine clones were identified, four of which possessed *in vivo *mammary outgrowth potential and five of which lacked *in vivo *outgrowth potential. Two of the clones formed mammary lobuloalveolar structures that contained both ducts and alveoli and were termed multipotent. Two of the clones generated either ductal-only or alveolar-only structures and were referred to as ductal-limited or alveolar-limited progenitor clones, respectively. The ability to expand the clones *in vitro *allowed for the characterization of their unique molecular phenotypes. Among the mammary-specific markers tested, high cytokeratin 5 (CK5) expression was the only marker that correlated with the clones' outgrowth potential. Among the clones that did not show any *in vivo *outgrowth potential when transplanted alone, one clone showed *in vivo *growth and incorporated into the mammary lumen when mixed with normal mammary epithelial cells. This clone also showed the highest *in vitro *expression of CK8 and *Elf5*and may represent a luminal-restricted progenitor clone. In addition, six "biclones," each made from an SP cell plus an NSP cell, were analyzed. Of these six, three exhibited lobuloalveolar growth.

**Conclusions:**

Distinct immortalized mammary progenitors have been isolated and characterized. Importantly, the results of this study provide further evidence for the existence of distinct ductal and alveolar mammary progenitors.

## Introduction

A mammary gland epithelial hierarchy is beginning to be defined. In 1996, Smith and colleagues [1] were the first to demonstrate, on the basis of limiting dilution transplantation studies, that the mouse mammary gland contained three distinct progenitors: lobular, ductal and lobuloalveolar. Later, Wagner and colleagues [2] discovered a parity-identified mammary epithelial subpopulation that was defined as a lobular-restricted progenitor cell. This cell type was also found in the luminal cell compartment of ducts, but was not a ductal progenitor cell. Although previous studies demonstrated the presence of these progenitors, their phenotypic characteristics were not known until now. Recently, flow cytometry (FACS) cell sorting followed by transplantation has allowed the phenotypic and functional characterization of progenitor and differentiated cells in mouse mammary glands and human breast cells. In the mouse mammary gland, cell surface markers have been used to define stem cells as lineage-negative (Lin^-^) CD24^+^/CD29^high ^[3] and Lin^-^CD24^+^/CD49f^high ^[4], luminal progenitors as Lin^-^CD29^low^CD24^+^CD61^+ ^[5], differentiated estrogen receptor-positive (ER^+^) luminal cells as CD24^+^/CD133^+ ^[6] and myoepithelial progenitors as CD29^low^/CD24^low ^[6]. In human breast cells, epithelial cell adhesion molecule-low (EPCAM^low/-^)/CD49f^high ^and EPCAM^+^/CD49f^+^/CD10^+^/Thy-1^+ ^represent bipotent progenitors that generate both luminal and myoepithelial cells [7,8], and EPCAM^+^/CD49f^+^/AC133^+^/MUC-1^+ ^represent luminal progenitor cells [9]. The differentiated luminal cells in human breast cells are characterized by EPCAM^+^CD49f^-^CD133^+^/MUC-1^+ ^expression, while the differentiated myoepithelial cells are characterized by EPCAM^+^/CD49f^-^/CD10^+^/Thy-1^+ ^expression [9]. However, the distinct progenitors have not been prospectively isolated, expanded *in vivo *or *in vitro *or fully characterized. Using single-cell cloning, distinct mammary progenitor populations were isolated. The CD cell line was originally derived from midpregnant BALB/c mouse mammary glands [10]. This cell line is unique in that transplantation of cells into the epithelium-free fat pads of syngeneic female mice generates mammary ductal and alveolar structures. The CD cell line harbors two distinct p53 mutations: (1) a G-to-C transversion resulting in substitution of tryptophan for cysteine at codon 138 and (2) a deletion of the first 21 nucleotides of exon 5 resulting in deletion of codons 123 to 129 [11]. The experiments in this study were performed using COMMA-D cell line engineered to express β-galactosidase (CDβ) cells. CDβ cells were derived from the parental CD cells by transduction with Zeg^+ ^retrovirus containing a bifunctional LacZ/Neomycin (β-geo) generated in Dr Soriano's laboratory [12,13]. Detailed sequence analysis of the *p53 *gene in eight different clonal derivatives of the CDβ cell line showed that both mutant alleles were present in each clone, demonstrating that the CD cell line is indeed clonal with respect to the *p53 *gene and that each cell expresses two distinctive mutant alleles of *p53 *[11]. As a result of these *p53 *mutations, the mammary outgrowths progress to mammary tumors after many months *in vivo*. Here we show that single-cell cloning of CDβ cells resulted in isolated ductal-limited, alveolar-limited and lobuloalveolar mammary progenitors. Molecular characterization of these clones using FACS- and immunofluorescence (IF)-defined markers specific for mammary progenitor activity. In summary, the progenitor clones derived from the CDβ cell line provide valuable tools for molecular characterization of multipotent and unipotent or committed mammary progenitors. Furthermore, the malignant potential of the CDβ clones will allow the study of the role of distinct progenitors in generating heterogeneous mammary tumors.

## Materials and methods

### Single-cell cloning

CDβ cells grown in Dulbecco's modified Eagle's medium/Ham's F-12 nutrient mixture containing 2% adult bovine serum albumin, 5 ng/ml epidermal growth factor, 5 μg/ml insulin and 100 U/ml penicillin/streptomycin were trypsinized, and a single-cell suspension at 1 million cells/ml was prepared. The process for mammary cell staining using Hoechst 33342 dye (B2261, St. Louis, MO, USA) has been published previously [14]. The Hoechst dye was excited at 350 nm and was fluorescence measured at two wavelength emissions of 450/20 band pass filter (Hoechst blue) and 675 EFLP optical filter (Hoechst red). Analysis and sorting were performed on a triple laser MoFlo (Cytomation, Fort Collins, CO, USA). After analysis, single cells from each side population (SP) and non-side population (NSP) region were sorted into individual wells of 96-well plates. For the biclones, one cell from the SP region and one cell from the NSP region were sorted into the same well.

### Fat pad transplantation and pituitary isografts

Mammary epithelial clearance, transplantation procedures and pituitary isograft procedures have been described previously [15,16]. These procedures were approved by the Institutional Review Board at Baylor College of Medicine, Houston, TX, USA. One hundred thousand cells from each clone were injected into the cleared fat pads of syngeneic (BALB/c) female mice. Eight weeks after cell transplantation fat pads were excised and prepared for the various staining procedures described in this section.

### Whole mount, hematoxylin and eosin and X-gal staining of mammary outgrowths

#### Whole-mount staining

Mammary glands were removed and fixed in 10% formalin for 24 hours. Fixative was removed, and tissue was placed in acetone for two incubations of 24 hours each. Glands were pretreated for 1 hour each in 100% and 95% alcohol, followed by staining overnight in hematoxylin. The next morning glands were immersed in running tap water for 1 hour, followed by dehydration in graded alcohol for 1 hour each at 70%, 95% and 100% (three times), as well as in xylene (three times). The tissues were kept in methylsalicylate for long-term storage. For X-gal staining, glands were fixed in 4% paraformaldehyde, followed by three washes using a buffer containing 0.2% NP-40, 0.01% NaDOC and 2 mM MgCl_2 _in 1× phosphate-buffered saline (PBS) and incubated overnight at 37°C in X-gal solution containing 1 mg/ml X-gal. Tissues were then dehydrated as described above, embedded in paraffin and sectioned.

#### Hematoxylin and eosin staining

Excised mammary outgrowths were fixed in 4% paraformaldehyde and then embedded in paraffin according to established protocols. Slides containing 5-μm sections were deparaffinized in xylene (8400 laboratory grade, Anapath brand; StatLab, Lewisville, TX, USA) and hydrated through graded ethanol series (100%, 95%, 80% and 70%), followed by hematoxylin staining (s212A Harris hematoxylin with glacial acetic acid; Poly Scientific (Bayshore, NY, USA) for 30 seconds and destaining in acid ethanol (1 ml of concentrated HCl + 400 ml of 70% ethanol), followed by eosin staining (176 Eosin Phloxine stain; Poly Scientific) for 45 seconds and dehydration in graded ethanol (95% and 100%) and xylene three times for 15 minutes each, followed by placement of a coverslip onto the slide by using xylene-based Permount (SP15-100 histological mounting medium, Fisher Scientific, Pittsburgh, PA, USA).

### Immunofluorescence and antibodies

IF was performed following tissue deparaffinization by clearance in xylene and hydration through a graded ethanol series as described above. Microwave antigen retrieval (20 minutes) in 10 mM sodium citrate was performed for all the antibodies used for IF. A 5% solution of bovine serum albumin (BSA) in PBS + 0.5% Tween 20 was used as blocking buffer. For staining of cells grown in two dimensions (2D), cells were grown on coverslip slides and fixed in 2% paraformaldehyde for 15 minutes at room temperature without antigen retrieval. Sections were incubated with the following primary antibodies overnight at 4°C: ERα rabbit polyclonal antibody (SC-542, MC-20; Santa Cruz Biotechnology, Santa Cruz, CA, USA), smooth muscle actin (SMA) antibody (A14, A2547; Sigma, St. Louis, MO, USA), cytokeratin 5 (CK5) rabbit polyclonal antibody (PRB-160P; Covance, Richmond, CA, USA), CK6 rabbit polyclonal antibody (PRB-169P; Covance), CK8 rat monoclonal antibody (TROMA-1; Developmental Studies Hybridoma Bank, University of Iowa, Iowa City, IA, USA), Na^+^, K^+^, 2Cl^- ^type I cotransporter (NKCC1) rabbit polyclonal antibody (gift of Jim Turner, National Institutes of Health, Bethesda, MD, USA) and p63 mouse monoclonal antibody (MS-1081-P, Thermo Fisher Scientific, Fermont, CA, USA). Nuclei were counterstained with 4',6-diamidino-2-phenylindole (Dapi) (Vector Laboratories, Burlingame, CA, USA) and TO-PRO-3 iodide (T3605, Invitrogen, Carlsbad, CA, USA). Secondary antibodies used were anti-rabbit Alexa Fluor 563 antibody, anti-mouse Alexa Fluor 594 antibody and anti-rat Alexa Fluor 488 antibody (Invitrogen, Carlsbad, CA, USA). All primary antibodies were used at a 1:200 concentration, and secondary antibodies were used at a 1:500 concentration. Confocal microscopy was performed using a laser-scanning confocal microscope (model 510; Carl Zeiss MicroImaging, Inc., Thornwood, NY, USA). The acquisition software used was LSM Image Browser (Carl Zeiss MicroImaging, Inc.). Phase-contrast images were captured using an inverted microscope (CK40-SLP; Olympus, Southend-on-Sea, Essex SS2 5QH, UK). The acquisition software used was PhotoShop version 5.0 (Adobe, San Jose, CA, USA).

### FACS and reagents

Primary antibodies used were anti-mouse CD29 fluorescein isothiocyanate (FITC)-conjugated antibody (102206; Biolegend, San Diego, CA, USA), anti-mouse CD24 R-phycoerythrin (PE)-conjugated antibody (553262; BD Biosciences, San Diego, CA, USA), anti-mouse CD24 FITC-conjugated (553261; BD Biosciences), anti-mouse CD49f FITC-conjugated antibody (557510; BD Biosciences), biotin-conjugated hamster anti-mouse CD61 antibody (553345; BD Biosciences), anti-stem cell antigen 1 (Sca-1)-PE antibody (553336; BD Biosciences) and anti-mouse prominin 1 antibody (AC133, 12-1331; eBioscience (San Diego, CA, USA). Biotin-conjugated CD61 was labeled using streptavidin-APC (554067; BD Biosciences). Isotype control staining was performed using PE-conjugated anti-rat immunoglobulin G2a (IgG2a) antibody (558067; BD Biosciences) and FITC-conjugated mouse anti-rat IgG1 antibody (553892; BD Biosciences). JSE3, 33A10, 50B8 and 44G3 antibodies were supernatants used at 1:1 concentration for 45 minutes at room temperature. All other antibody cells were stained at a final concentration of 1:200 for 30 minutes on ice, followed by washes in Hanks' balanced salt solution (Invitrogen, Carlsbad, CA, USA) containing 2% fetal bovine serum. FACS analysis and data acquisition were performed using the BD LSR II flow cytometer and BD FACSDiva-based software (BD Biosciences).

### Intracellular staining and FACS analysis

Cells were harvested by trypsinization with 0.05% trypsin-ethylenediaminetetraacetic acid (Invitrogen). Cells were fixed for 15 minutes with Click-iT fixative (Invitrogen), permeabilized for 30 minutes with saponin-based permeabilization buffer (Invitrogen) and stained with primary CK5 antibody (1:50, PRB-160P; Covance) or with the rabbit IgG immunoglobulin isotype control antibody (BD Biosciences). Subsequently, samples were counterstained with Alexa Fluor 488-conjugated goat anti-rabbit secondary antibody (1:100; Invitrogen) and resuspended in 300 μl of 1% BSA-PBS prior to analysis with the LSR II flow cytometer. Data analysis was performed using FlowJo software (Tree Star, Inc., Ashland, OR, USA).

### RNA isolation and quantitative real-time polymerase chain reaction

Total RNA was isolated and reverse-transcribed (100 ng) using the TaqMan Gene Expression Cells-to-CT Kit (Ambion, Austin, TX, USA) following the manufacturer's instructions. The mRNA transcript levels of the housekeeping gene *18S *and the target gene *Elf5 *were evaluated using commercially available TaqMan primers and probes (Hs03928990_g1, *18S*; Mm00468732_m1 *Elf5 *(Applied Biosystems, Carlsbad, CA, USA)). The resulting cDNA was diluted 1:15 and subjected to quantitative real-time polymerase chain reaction (qRT-PCR) analysis in a 20-μl final reaction volume. Each sample was run in triplicate for both the target and the normalizer, and the average cycle threshold (*C*_t_) was used in subsequent calculations. Each sample was also run as a minus reverse transcriptase control to confirm lack of DNA contamination. Gene expression was detected using the ABI 7900 fast real-time PCR system (Applied Biosystems). The 2-ΔΔ^*C*t ^method was used to calculate relative fold change values between samples, with one control sample set to 1 and all other samples compared to it.

## Results

### Single-cell cloning of CDβ cells identified multipotent and committed progenitors

To assess the stem progenitor cell potential of the SP cells compared to the NSP cells, CDβ cells were stained using Hoechst dye 33342, followed by FACS analysis and sorting of each population. Cells that effluxed the dye appeared on the left side of a FACS analysis panel and were referred to as SP cells. Cells that retained the dye appeared on the right side and were referred to as non-NSP cells. Procedures for isolation of SP and NSP mammary cells have been described in detail previously [14,17]. Five thousand sorted cells from each region were transplanted into cleared fat pads of syngeneic female mice and evaluated for outgrowth potential 6 weeks following transplantation. The results showed that there was no difference in the outgrowth potential of SP vs. NSP cells derived from the CDβ parental cells (Additional file 1). The outgrowth potential (take rate) was calculated by dividing the number of fat pads containing positive mammary outgrowths by the total number of fat pads transplanted. Because there was no difference in repopulation ability between the SP and NSP cells, single cells were sorted from each region, expanded *in vitro *and transplanted to assess their outgrowth potential. In addition, biclones were made by adding one SP cell to one NSP cell, expanded *in vitro *and transplanted. One hundred thousand cells from 15 selected groups were transplanted into cleared fat pads of syngeneic female mice to evaluate their outgrowth potential. Eight weeks following transplantation fat pads were excised and examined for the presence of mammary outgrowths by whole-mount staining. If transplantation of cells gave rise to distinguishable ductal and/or alveolar structures, the groups were stated to possess outgrowth potential. However, if the groups did not show any *in vivo *growth or showed growth consisting of no discernible mammary structures, they were referred to as nonprogenitor populations, indicated by the "No outgrowth" designation in Table 1, column 3. Groups 1 to 6 were designated as biclones, since they were derived from two cells, one each from the SP and NSP regions. We sorted and combined two cells to examine whether the presence of both cell types (SP and NSP) was required to generate an outgrowth. However, as seen in Table 1, outgrowths were generated from clones that arose from single SP or NSP cells. Clones 7 to 10 were derived from single cells sorted from the SP region, and clones 11 to 15 were derived from single cells sorted from the NSP region (Table 1). All 15 groups were evaluated by transplantation to assess their outgrowth potential. The biclones were not evaluated beyond outgrowth potential. Seven of the 15 groups gave rise to mammary outgrowth (Table 1). The percentage of fat pads filled and the fraction of positive outgrowths are indicated. Three of five NSP-derived clones had outgrowth potential compared to one of the four SP-derived clones. Transplantation of clone 9 generated only alveolar structures *in vivo *and is referred to herein as an alveolar progenitor. Transplantation of clone 12 generated only ductal structures and is referred to herein as a ductal progenitor. Transplantation of clones 11 and 15 generated ducts and alveolar structures and are referred to herein as multipotent progenitor populations. Clone 13 incorporated into the mammary lumen when mixed with unsorted mammary epithelial cells prior to transplantation and is referred to herein as a luminal restricted progenitor. The outgrowth take rate for the parental CDβ cells injected at 100,000 cells/fat pad is 100% (six of six; data not shown).

**Table 1:** Transplantation results of the CDβ-derived clones^a^

Clone	Source	Progenitor potential	Outgrowth rate	Number of fat pads filled, *n *(%)
1	Bi	-	6/12	6 (50%)
2	Bi	-	0/12	No outgrowth
3	Bi	-	6/8	4 (25%)
				2 (75%)
4	Bi	-	7/7	
5	Bi	-	0/9	No outgrowth
6	Bi	-	0/11	No outgrowth
7	SP-1	-	0/12	No outgrowth
8	SP-2	-	0/12	No outgrowth
9	SP-3	Alveolar	12/12	6 (50%)
				6 (25%)
10	SP-4	-	0/10	No outgrowth
11	NSP-1	Multipotent	12/12	6 (75%)
				6 (50%)
12	NSP-2	Ductal	7/10	6 (75%)
				1 (25%)
13	NSP-3	Luminal	0/6	No outgrowth
14	NSP-4	-	0/3	No outgrowth
15	NSP-5	Multipotent	5/8	2 (75%)
				3 (25%)

^a^CDβ = COMMA-D cell line engineered to express β-galactosidase; No outgrowth = no discernible mammary structures; SP = side population; NSP = non-side population; Bi = biclonal, referring to the groups derived from a single SP cell and a single NSP cell. The groups were either derived from a single SP cell or a single NSP cell (clones 7 to 15) or were biclonal and derived from one SP mixed with one NSP (clones 1 to 6). Transplantation of clone 9 or SP-3 generated alveolar-only structures *in vivo *and was referred to as the alveolar progenitor clone. Transplantation of clone 12 or NSP-2 generated ductal-only structures and was referred to as the ductal progenitor clone. Clones 11 (NSP-2) and 15 (NSP-5) generated ducts and alveolar structures and were referred to as multipotent. Clone 13 or NSP-3 incorporated into the lumen when mixed with total mammary epithelial cells prior to transplantation and was referred to as luminal. Outgrowth take rate was calculated by dividing the number of positive fat pads containing any mammary outgrowths divided by the total number of fat pads transplanted. The number of fat pads filled refers to the percentage of fat pads containing mammary outgrowths. The numbers outside the parentheses refer to the number of fat pads containing a mammary outgrowth. The percentages inside the parentheses refer to the percentage of fat pads containing a mammary outgrowth.

**Figure 1:**
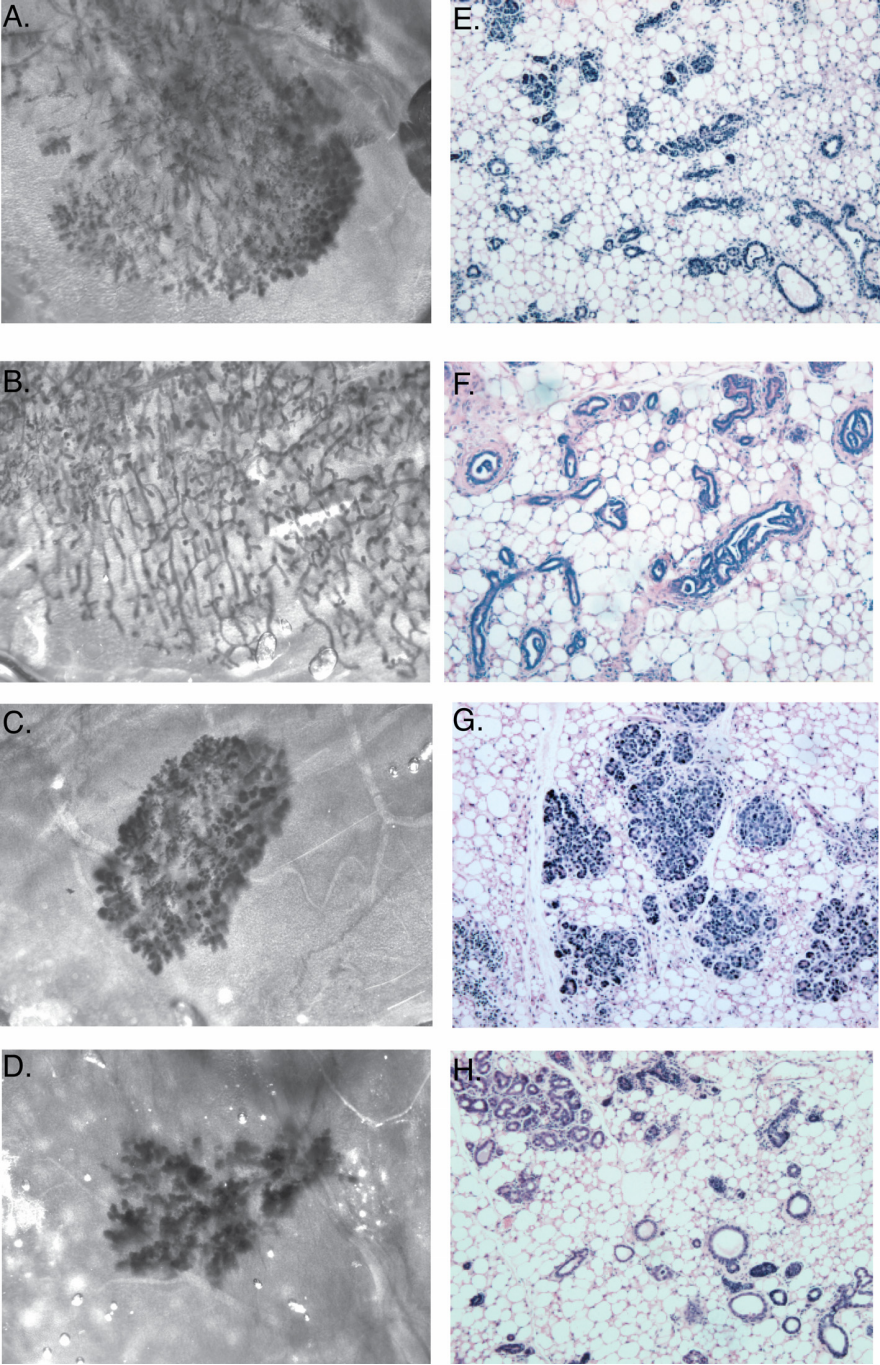
**Whole-mount and hematoxylin and eosin staining of outgrowths generated from the CDβ parental line and CDβ progenitor clones**. **(A and E) **Transplantation of COMMA-D cell line engineered to express β-galactosidase (CDβ) parental cell line generates outgrowths containing ductal and alveolar structures. **(B and F) **Ductal progenitor cells give rise to outgrowths containing ducts only. **(C and G) **Alveolar progenitor clone generates mainly alveoli and a limited number of small ducts. **(D and H) **Multipotent progenitor clone generates outgrowths containing ductal and alveolar structures. Figures 1A to 1D are whole-mount-stained images taken at ×3.2 original magnification. Figures 1E to 1H are hematoxylin and eosin-stained images taken at ×10 original magnification.

**Figure 2:**
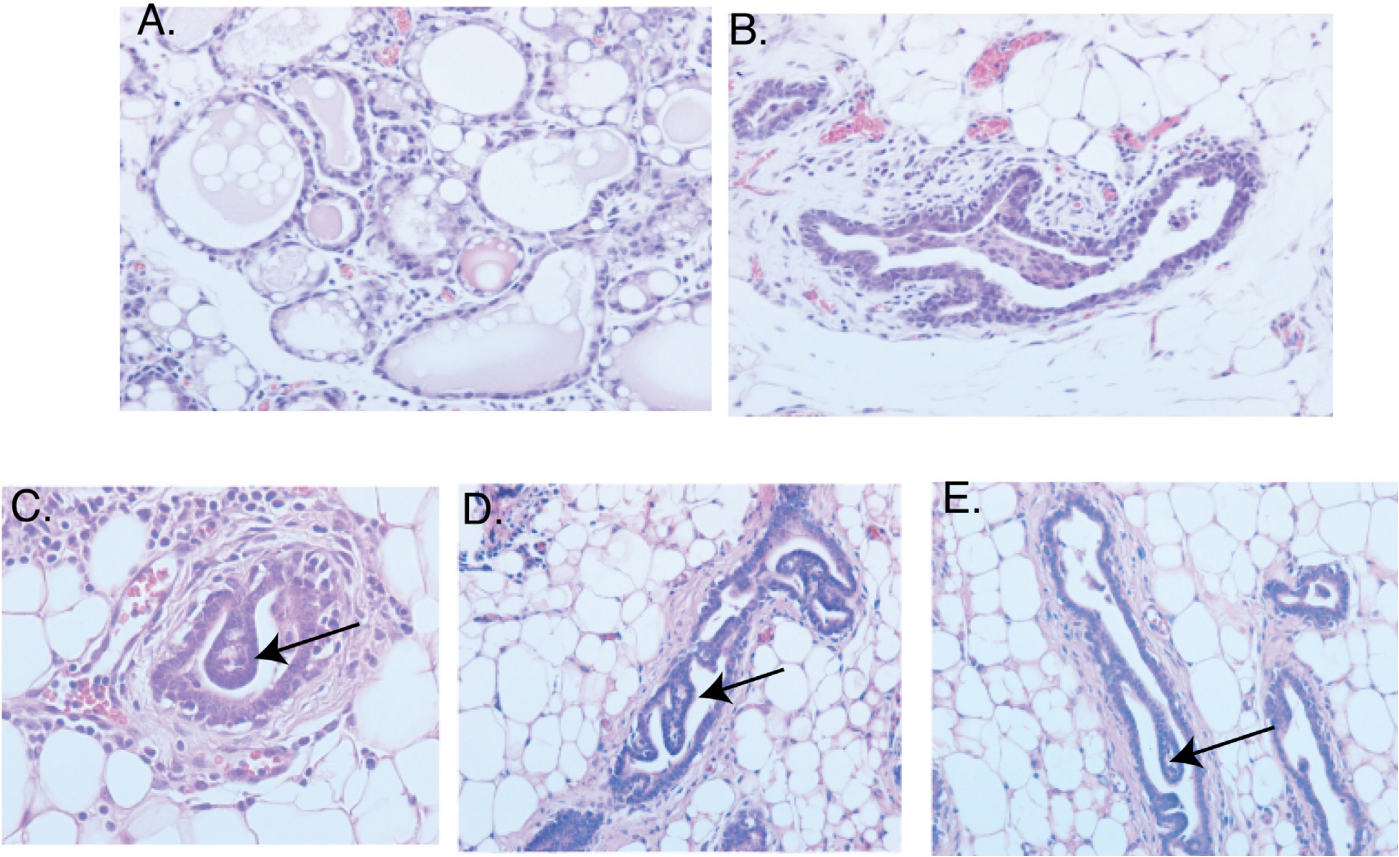
**Hematoxylin and eosin (H&E) staining of alveolar progenitor clone makes lipid droplets indicative of alveolar differentiation**. **(A) **Alveolar progenitor-derived outgrowths following hormonal stimulation using pituitary isografts show the formation of lipid droplets inside the lumen. **(B) **Ductal progenitor outgrowths, shown for comparison, are incapable of alveolar differentiation, and the ducts lack the ability to form lipid droplets. **(C to E) **Intraductal papillary hyperplasia (IDH) generated by the ductal progenitors. Arrows indicate IDH lesions. H&E images taken at ×20 original magnification.

Figure 1 shows whole mounts (Figures 1A to 1D) and hematoxylin and eosin staining (Figures 1E to 1H) of outgrowths generated by the selected clones. As shown, outgrowths generated from the parental CDβ cells consisted of ductal and alveolar structures (Figures 1A and 1E). In contrast, one of the clones referred to as a ductal progenitor generated only ducts (Figures 1B and 1F), while another clone referred to as an alveolar progenitor generated alveoli with a very limited number of small ducts (Figures 1C and 1G). Two of the clones generated outgrowths consisting of ducts and alveoli and were referred to as multipotent. Figures 1D and 1H are an outgrowth generated from one of the multipotent progenitor clones. Mice transplanted with cells from the alveolar progenitor (Figure 2A) and the ductal progenitor (Figure 2B) clones were exposed to prolactin, estrogen and progesterone using pituitary isografts. Despite hormonal stimulation and subsequent retransplantation, no alveolar differentiation was evident in any of the ductal progenitor-derived outgrowths. These data demonstrate that the ductal progenitor clone is devoid of alveolar differentiation potential. In contrast, the alveolar progenitor-derived outgrowths consisted of alveoli and a limited number of small ducts. As shown in Figure 2A, alveolar progenitor-derived outgrowths, with hormonal stimulation, are capable of producing large lipid droplets, a marker of alveolar differentiation. Cells from the alveolar progenitor clone grown on Matrigel™ basement membrane matrix (BD Biosciences, San Diego, CA, USA) were also capable of expressing β-casein (data not shown). Interestingly, as shown in Figures 2C to 2E, the ducts generated from the ductal progenitor clone showed loss of normal directional cues and formed intraductal papillary hyperplasia (IDH). About 10% to 50% of the ducts showed this IDH phenotype. The appearance of IDH lesions was independent of hormonal stimulation and was seen in both virgin and hormonally stimulated transplants. The reason for this abnormal growth pattern is not known and may be related to a deficiency in guidance cues imposed by other missing cell types or by genetic mutations.

### Distinct progenitors generate outgrowths with appropriate luminal or basal orientation

**Figure 3:**
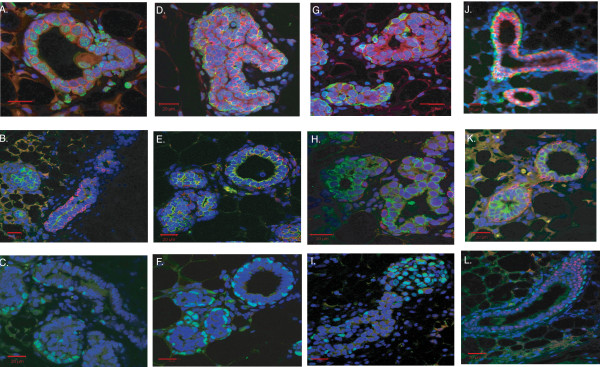
**Immunofluorescence (IF) staining of CDβ-derived progenitor clones showing appropriate basal or luminal orientation**. **(A to C) **CDβ multipotent outgrowths. **(D to F) **Ductal-limited outgrowths. **(G to I) **Alveolar-limited outgrowths. **(J to L**) Adult primary mammary outgrowths (primary BALB/C). **(A, D, G and J) **Na^+^, K^+^, 2Cl^- ^type I cotransporter (NKCC1) (red) and mouse smooth muscle actin (SMA) (green). **(B, E, H and K) **Cytokeratin 5 (CK5) (red) and CK8 (green). **(C, F, I and L) **p63 (green) and nuclear estrogen receptor (ER) (red). The nuclei were counterstained with 4', 6-diamidino-2-phenylindole and TO-PRO-3 iodide.

The expression of mammary-specific basal markers p63, CK5 and SMA, as well as luminal markers CK8, NKCC1 and ER, was examined using IF in outgrowths generated from the distinct progenitor clones. These studies showed that the majority of outgrowths contained all the mammary cell types in the correct luminal or basal orientation (Figures 3A to 3L). In most of the outgrowths, there was appropriate basal expression of SMA, CK5 and p63 (Figures 3A to 3L) and luminal expression of CK8 and NKCC1 (Figures 3A to 3K). As described earlier regarding Figure 2, the outgrowths generated by transplantation of the ductal progenitor clone showed a normal ductal phenotype as well as an IDH phenotype. The latter phenotype resulted in the expression of CK5, the basal marker, inside some of the disorganized ducts (data not shown). These results demonstrate a model of mammary development where ductal and alveolar progenitors are independently capable of generating luminal as well as basal cell types.

As shown in Figures 3C, F, I and 3L, nuclear ER expression was undetectable in the CDβ-derived outgrowths, although it was readily detectable in the normal BALB/c mammary glands (Figure 3L). This was surprising, since many studies have reported the importance of ER expression for normal mammary development [18,19]. However, studies using mouse models with complete germline deletion of ER (ER^-^/^-^) have shown that ER was not required for prepubertal mammary development, since at 3 weeks of age there was no difference between the mutant and wild-type littermates in the formation of a rudimentary mammary ductal tree [19]. Nevertheless, there was a significant difference in ductal elongation postpuberty in female mice lacking ER [19]. Furthermore, Medina and colleagues [20] characterized *p53*-null preneoplastic mammary outgrowth lines which lacked ER expression but retained normal ductal alveolar outgrowth potential. The outgrowths were immortal and progressed through IDH and carcinoma over time. Therefore, ER may not be required for mammary development in the CDβ-derived outgrowths, since they also lack wild-type *p53 *gene expression. The outgrowths were also examined for the expression of progesterone receptor (PR). PR expression was solely extranuclear in the outgrowths generated from all ductal, alveolar and multipotent progenitor clones (data not shown). Interestingly, extranuclear PR expression has been implicated in the activation of Src and mitogen-activated protein kinase (MAPK) that lead to cancer cell proliferation [21]. These interactions may also mediate hyperplastic growth of CDβ-derived outgrowths and their progression to tumorigenesis.

### High CK5 expression correlates with outgrowth potential

CDβ progenitor clones were examined for the expression of specific CKs and surface markers known to be expressed by mammary stem, progenitor and differentiated cells grown in 2D. These included markers of mouse mammary repopulating cells (CD24^+^/CD29^high ^and CD24^+^/CD49f^high^), luminal ER-positive (CD24^+^/AC133^+^), Sca-1 (hematopoietic stem progenitor cell marker) and luminal progenitors (CD29^low^/CD24^+^CD61^+^) [3-6,22]. Other surface markers analyzed included JSE3, 33A10, 50B8 and 44G3 (monoclonal antibodies developed in the laboratory of Dr Around Sonnenberg [23]) and aldehyde dehydrogenase (Aldefluor Kit, Stem Cell Technologies, Vancouver, BC, Canada). Additionally, the expression of CK5, CK8, CK6, SMA, NKCC1, total p63, TA p63 and ΔN p63 were examined by IF staining on cells grown in 2D culture, and additional staining was performed to analyze P63, TA p63, ΔN p63 and CK5 expression by FACS. *P63 *is a member of the *p53 *family. It is critical in the development of stratified epithelial tissues such as epidermis, breast and prostate. *P63 *is expressed in at least six isoforms that can be classified into two groups: those with a transactivating domain (TAp63) and those that lack this domain (ΔNp63) [24].

**Figure 4:**
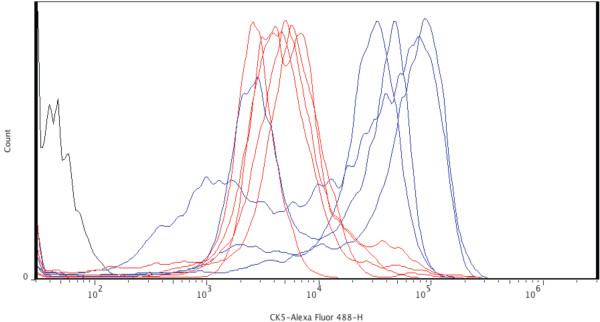
**CK5 is uniquely expressed by the clones that possess outgrowth potential**. The intensity of CK5 expression was assessed by FACS using a CK5-specific antibody conjugated to Alexa Fluor 488. The histogram shows the intensity of CK5 expression in clones devoid of outgrowth (red) (clones 1, 8, 10, 13 and 14) compared to the clones that formed outgrowths *in vivo *(blue) (alveolar, ductal and multipotent progenitors). The isotype control is shown in black.

IF studies for intracellular markers were done by counting an average of about 500 cells/marker. These studies showed that among the markers tested, only high expression of CK5 correlated with a clone's outgrowth potential (Figure 4). As shown, the three progenitor clone types, multipotent, ductal and alveolar, expressed CK5 with a mean fluorescence intensity (MFI) of 1 × 10^4.5 ^(pink histograms). The clones that showed no *in vivo *outgrowth potential showed low-intensity expression of CK5 with a MFI of 1 × 10^3.7 ^(blue histograms). Interestingly, the alveolar progenitor and ductal progenitor clones showed two peaks: a low-intensity peak (blue histogram) and a high-intensity peak (pink histogram). The reason for the high-intensity vs. low-intensity peak expression of CK5 may be related to the existence of a heterogeneous population of luminal and basal cells. The expression of CK5 by the subpopulation of cells representing mammary repopulating activity (Lin^-^CD24^+^CD29^high^) has been proposed [25-27]. Furthermore, Deugnier and colleagues [28] showed that Sca-1^high ^cells in the CDβ cell line possessed significantly higher morphogenic potential and contained a significantly higher percentage of cells expressing the basal markers CK5 and p63 compared to Sca-1^-/low ^cells (about 95% vs. 5%). The molecular mechanism underlying CK5 expression in stem and progenitor cells is not clear at this point and requires further investigation.

No correlation could be found between the expression of CK8, NKCC1, CK6, SMA, total and isoform-specific (TA and ΔN) p63 and Aldefluor and progenitor activity (data not shown). Our data show that Sca-1 and p63 expression levels change *in vitro *in cells grown in 2D, and therefore their expression could not be correlated with the clones' outgrowth potential (data not shown). Therefore, on the basis of our data, Sca-1 expression may not be correlated with mammary progenitor potential. The expression of individual p63 isoforms, TA and ΔN, by IF and FACS (ΔN and TA), also was not associated with *in vivo *progenitor potential (data not shown).

### Restricted mammary luminal progenitor cells may exist in the mouse mammary gland

**Figure 5:**
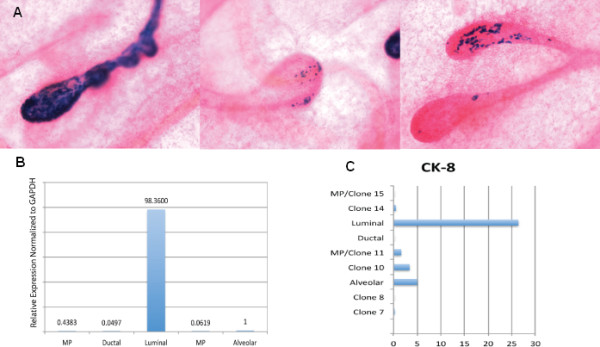
**Luminal-restricted clone incorporates into the ductal lumen *in vivo *and expresses significantly higher luminal-specific markers CK8 and Elf5**. **(A) **The luminal-restricted clone incorporated into the ductal lumen when mixed with primary mammary epithelial cells prior to transplantation. The luminal-restricted cells stained blue upon X-gal staining, since they were derived from CD cells previously engineered to express β-gal. **(B) **Expression of Elf5 by quantitative real-time polymerase chain reaction is significantly higher in the luminal-restricted clone compared to the remaining progenitor clones. GAPDH, glyceraldehyde 3-phosphate dehydrogenase. **(C) **Higher percentage of cells in the luminal-restricted clone expressed CK8 compared to the other progenitor clones and nonprogenitor clones 7, 8, 10, and 14. CK8 expression was measured by IF using specific antibody. An average of 500 cells were counted in each group. The *x*-axis represents the percentage of CK8-positive cells divided by the total number of cells counted. On the *y*-axis, MP/clone 11 refers to multipotent clone 11 and MP/clone 15 refers to multipotent clone 15.

Clone 13, referred to as the luminal-restricted progenitor, did not show any *in vivo *outgrowth potential, but incorporated into the ductal lumen when coinjected with normal mammary epithelial cells (Figure 5A). The mixing experiment was done because the luminal progenitor clone was derived from β-gal-expressing CDβ cells and would be distinguished from the normal mammary epithelial cells by X-gal staining. As expected, the luminal progenitor clone expressed significantly higher CK8 (a mammary luminal marker) by IF and Elf5 (a luminal progenitor marker) by qRT-PCR (Figures 5B and 5C). On the basis of these results, we propose that this clone represents a luminal-restricted progenitor clone.

## Discussion

A role for stem cells or distinct progenitor cells as the cells of origin in many types of cancer has been proposed [29-32]. These studies have led to the hypothesis that in many types of cancers, including breast cancer, distinct progenitors and their abrogated self-renewal pathways may ultimately underlie the mechanisms of heterogeneity observed among subtypes of human breast cancer [33]. The identification and further characterization of distinct mammary progenitors are necessary prerequisites for testing this hypothesis. Single-cell cloning of an immortalized mammary cell line, CDβ, was utilized to isolate mammary-specific multipotent, ductal and alveolar progenitor clones. Molecular phenotyping showed that mammary progenitors express significantly higher levels of CK5.

Sca-1 expression was not associated with outgrowth potential of the clones. The unique expression of Sca-1 in stem and progenitor subpopulations has been proposed for many cell types. Sca-1 (Ly-6A), a member of *Ly6 *gene family, is a glycosylphosphatidylinositol-anchored cell surface protein. Sca-1, when combined with other markers, c-kit and the hematopoietic lineage, identifies hematopoietic stem cells in the bone marrow [34]. However, its role as a stem or progenitor cell marker in the mammary gland has been controversial [35].

CDβ clones that possess outgrowth potential contain significantly higher levels of CK5 expression. The expression of CK5 in mammary stem cells has been proposed previously [25-27]. In human breast tissue, there is a small population of CK5-expressing cells (5%) that lack the expression of other markers of glandular, CK8, CK18, CK19 or myoepithelial cells (SMA) [25]. Additionally, the stem cell subpopulation in normal mouse mammary gland (Lin^-^CD24^+^CD29^+^) is enriched in the expression of CK5 and displays a basal phenotype [27,36]. Dontu and colleagues [26] showed that mammospheres were enriched in undifferentiated cells and expressed the basal markers CK5 and CK14. These data demonstrate that high-level CK5-expressing cells may represent an enriched population of stem and progenitor cells. It will be interesting to explore whether CK5 serves only as a stem cell marker or perhaps is coregulated with a critical stem cell gene.

In light of the important role of estrogen signaling during normal mammary development, it was surprising that the CDβ-derived outgrowths lacked ER expression. This may be because the CDβ-derived outgrowths express mutated *p53*. It has been demonstrated that some of the *p53*-null preneoplastic mammary outgrowths lack ER expression, although they do develop normally [37]. Therefore, the requirement for ER expression may apply only to wild-type *p53*-expressing mammary glands. Furthermore, ER^-^/^- ^mammary glands develop normally until puberty. However, there is stunted ductal outgrowth after puberty. Therefore, the outgrowths generated by the CDβ-derived clones may develop without the expression of ER because the CDβ cells lack a functional *p53 *gene and/or the outgrowths grow in response to prepubertal growth hormones such as the parathyroid hormone signaling pathway [18]. The progesterone signaling pathway has also been shown to play an important role in mammary ductal branching morphogenesis and alveolar development, as well as in mammary tumorigenesis [18,21]. Interestingly, the expression of PR was extranuclear in the outgrowths derived from the progenitor clones. It has been demonstrated that PR may bind and activate Src-1 and MAPK signaling pathway and thus promote the proliferation of breast cancer cells. These extranuclear effects of PR may mediate mammary hyperplasia and tumor progression in the outgrowths derived from the CDβ cells.

The CDβ cell line is unique in that transplantation of cells into the epithelium-free fat pads of syngeneic female mice generates mammary outgrowths that eventually progress to tumors over time *in vivo*. Mammary tumors eventually form because the CDβ cell line harbors two distinct *p53 *mutations. Thus, the model provides a unique opportunity to study the cellular and molecular mechanisms underlying mammary tumor progression beginning at the normal stages.

**Figure 6:**
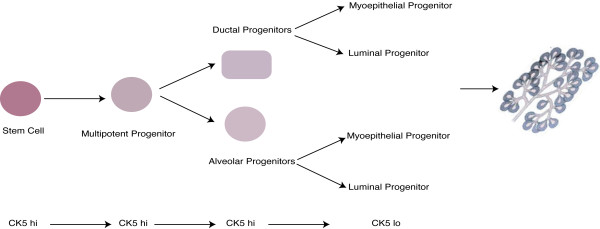
**Hypothetical model of stem cell differentiation during mammary gland development**. Mammary gland development begins by asymmetric self-renewal in a stem cell, which generates multipotent and bipotent ductal and alveolar progenitor cells. Ductal and alveolar progenitor cells are bipotent, express high CK5 levels and may give rise to luminal and myoepithelium-restricted progenitors. Upon commitment to transient amplification and differentiation, restricted progenitor cells may downregulate CK5 expression.

Figure 6 shows a hypothetical model of mammary development based on the findings in this study. As illustrated, mammary development begins by asymmetric self-renewal in a stem cell, which generates multipotent and bipotent ductal and alveolar progenitor cells. Each ductal and alveolar progenitor cell is bipotent, expresses high CK5 levels and may give rise to luminal- and myoepithelial-restricted progenitors. The luminal and myoepithelial progenitors give rise to the ductal and alveolar structures. Upon commitment to transient amplification and differentiation, restricted progenitor cells may downregulate CK5 expression.

## Conclusions

The utilization of clones with distinct multipotent, ductal, alveolar and restricted luminal progenitor activity will provide valuable tools for studying the unique biology of these progenitor populations. The identification and molecular characterization of self-renewal pathways in stem and distinct progenitor cell subpopulations will not only reveal normal mechanisms for development but also help to elucidate the molecular pathways that are misregulated during tumorigenesis, ultimately leading to tumor heterogeneity.

## Abbreviations

2D: two-dimensional; CDβ: COMMA-D cell line engineered to express β-galactosidase; CK: cytokeratin; FACS: flow cytometry; H&E: hematoxylin and eosin; IF: immunofluorescence; Lin: lineage; NKCC1: sodium potassium chloride cotransporter; NSP: non-side population; Sca-1: stem cell antigen 1; SMA: smooth muscle actin; SP: side population.

## Competing interests

The authors declare that they have no competing interests.

## Authors' contributions

FSK assisted in the study's conception and design, the collection and assembly of data, and data analysis and interpretation. MZC, SK, JH, WX, MZ and HLL assisted in the collection and assembly of data and in data analysis and interpretation. AS assisted in the provision of study material, the study's conception and design and data analysis and interpretation. JMR assisted in the study's conception and design, data analysis and interpretation, and financial support. DM assisted in the study's conception and design, data analysis and interpretation, and provision of study material. FB assisted in the study's conception and design, the collection and assembly of data, data analysis and interpretation, and manuscript writing. All authors read and approved the final manuscript.

## Supplementary Material

Additional file 1 **Transplantation results comparing SP, NSP and mixed (SP and NSP) cells sorted from CDβ cells**. CDβ cells sorted from each region representing SP, NSP, sorted and mixed SP and NSP showed similar outgrowth efficiency following *in vivo *fat pad transplantation of 5,000 cells. The *x*-axis of the bar graph refers to the fraction of fat pads containing positive outgrowths over the total number of fat pads transplanted in each sorted SP, NSP and mixed group. SP, side population; NSP, non-side population; CDβ, COMMA-D cell line engineered to express β-galactosidase. Click here for file
