# Supplementary figures and images for: Prospective isolation and characterization of committed and multipotent progenitors from immortalized mouse mammary epithelial cells with morphogenic potential
Source: Breast Cancer Res. 2011 Apr 5;13(2):R41. doi: 10.1186/bcr2863 (PMC3219204; doi:10.1186/bcr2863)

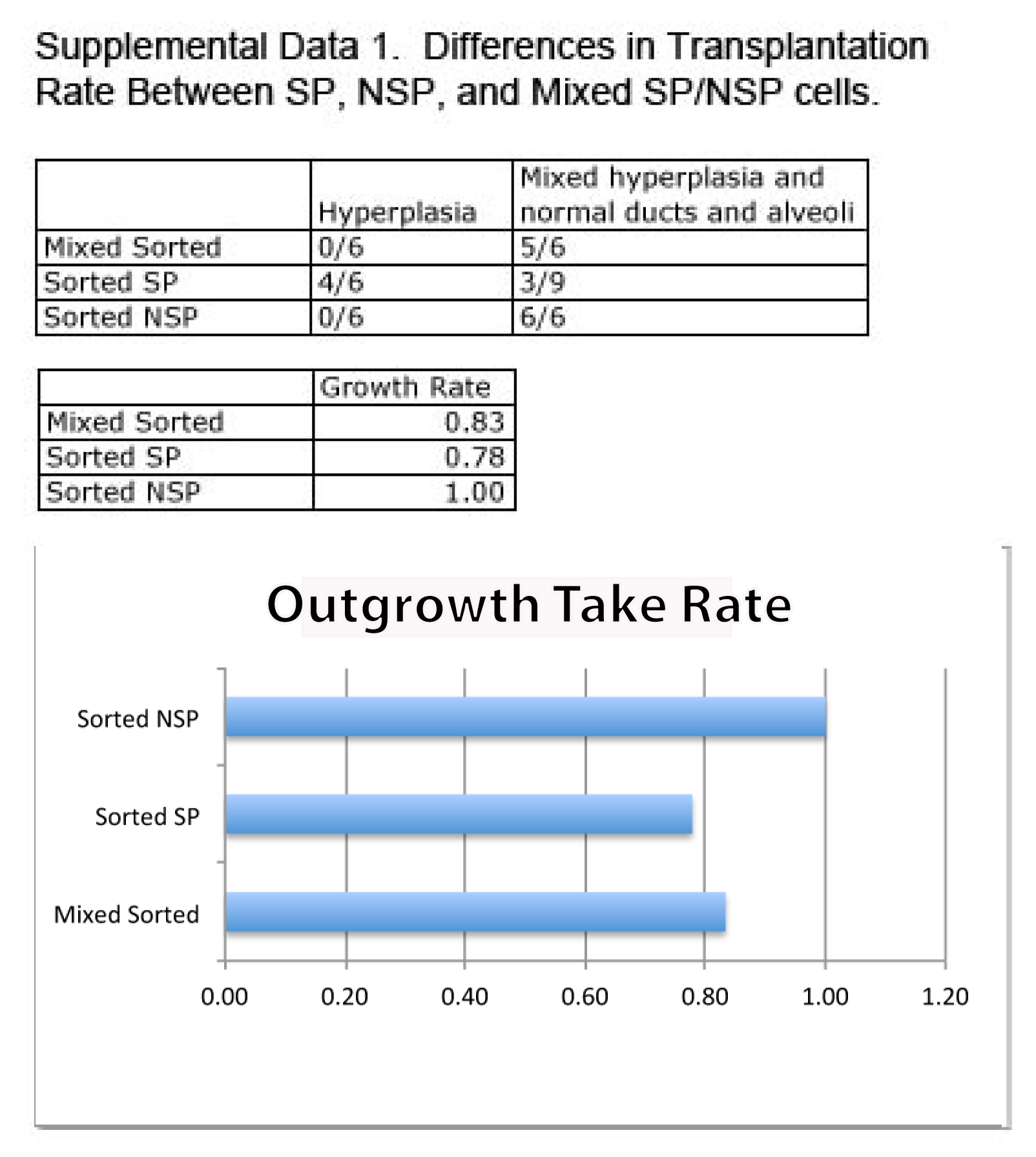

Supplement: Additional file 1 — Transplantation results comparing SP, NSP and mixed (SP and NSP) cells sorted from CDβ cells. CDβ cells sorted from each region representing SP, NSP, sorted and mixed SP and NSP showed similar outgrowth efficiency following in vivo fat pad transplantation of 5,000 cells. The x-axis of the bar graph refers to the fraction of fat pads containing positive outgrowths over the total number of fat pads transplanted in each sorted SP, NSP and mixed group. SP, side population; NSP, non-side population; CDβ, COMMA-D cell line engineered to express β-galactosidase. [file bcr2863-S1.PNG]
